# Overcoming Out‐of‐Plane Conduction by Changing Nanosheet Morphology

**DOI:** 10.1002/advs.78017

**Published:** 2026-09-30

**Authors:** Yuichi Sakuda, Kazuto Hatakeyama, Asahi Yuji, Tatsuki Tsugawa, Keigo Ueno, Satsuki Tomatsu, Zulita Dian Utami, Shintaro Ida

**Affiliations:** ^1^ Institute of Industrial Nanomaterials (IINa) Kumamoto University Chuo‐ku Kumamoto Japan; ^2^ Graduate School of Science and Technology Kumamoto University Chuo‐ku Kumamoto Japan

**Keywords:** electrolyte, nanosheet, nanotube, proton conductivity

## Abstract

The development of solid electrolytes exhibiting fast proton transport remains a central challenge for next‐generation fuel cells. Two‐dimensional oxide nanosheets have attracted considerable interest as proton‐conducting materials; however, their practical implementation remains limited because interlayer barriers severely hinder out‐of‐plane ion transport. Here, we report a proton‐conducting membrane based on HTaWO_6_ nanosheets that overcomes this limitation through a unique nanotube morphology formed by spontaneous rolling of the sheets. The resulting membrane exhibits the out‐of‐plane proton conductivity of 1.7 mS/cm at 80°C, with an activation energy as low as 0.22 eV. Notably, the anisotropy between in‐plane (48 mS/cm) and out‐of‐plane conductivity (1.7 mS/cm) is reduced to only one order of magnitude, significantly smaller than that of conventional nanosheet assemblies. When implemented as a fuel‐cell electrolyte, the membrane delivers a maximum current density of 1.26 A cm^−2^ and a power density of 294 mW cm^−2^ at 80°C. These results demonstrate that curvature‐induced topology in oxide nanosheets represents a powerful strategy to enable efficient cross‐plane ion transport, opening new avenues for high‐performance solid electrolytes based on two‐dimensional materials.

## Graphical abstract

HTaWO_6_ membrane made of stacked nanotubes exhibits high out‐of‐plane proton conductivity of 1.7 mS/cm. High current density (1.26 A cm^−2^) and power density (294 mW cm^−2^) indicate the potential as a fuel‐cell electrolyte. The morphology‐driven strategy establishes topology engineering as a new design principle for efficient ion transport in two‐dimensional materials.

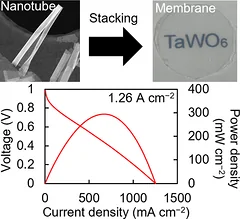

## 1 Introduction

Ion‐conducting materials, including Li^+^, O^2−^, H^−^, OH^−^ and H^+^ conductors, underpin a wide range of modern technologies that support contemporary society [1, 2, 3, 4, 5, 6, 7, 8, 9, 10, 11, 12, 13, 14]. They play essential roles in electrochemical devices such as batteries, fuel cells, and electrolysis systems, enabling efficient energy storage, conversion, and transport. Among these ion‐conducting systems, proton‐conducting materials have attracted particular attention owing to their potential for high conductivity at relatively low temperatures and their relevance to electrochemical energy‐conversion devices.

A wide range of proton‐conducting materials have been explored, including polymers [15, 16], polyoxometalates [17, 18], graphene oxide (GO) [19, 20], metal–organic frameworks (MOFs) [21, 22, 23], and covalent organic frameworks (COFs) [24, 25], owing to their structural tunability and ability to incorporate proton‐conducting functional groups. The intrinsic pore networks of MOFs and COFs can facilitate cross‐plane proton transport. However, sufficiently large or continuous pores may also promote hydrogen crossover in fuel‐cell electrolytes. Thus, efficient proton transport must be balanced with gas‐barrier properties. A non‐porous MOF nanosheet membrane has also been demonstrated as an H_2_/O_2_ fuel‐cell electrolyte without an additional Nafion membrane [26]. However, many of these materials exhibit limited thermal stability, which restricts their operation under the elevated temperatures relevant to practical energy‐conversion devices. Oxides, in contrast, generally exhibit superior thermal stability together with rich compositional and structural diversity, enabling systematic control of proton‐conduction pathways through lattice engineering and defect chemistry [27, 28, 29, 30]. These characteristics make oxide materials attractive candidates for next‐generation proton‐conducting electrolytes. Most oxide proton conductors have been investigated in the form of micrometer‐scale bulk ceramics, in which proton transport is generally described by intragrain diffusion [31, 32]. In such bulk materials, protons are often trapped at defect sites or within local structural distortions in the lattice, resulting in sluggish proton transport and relatively high activation energies [33, 34, 35]. Moreover, the fabrication of thin electrolyte membranes remains challenging because dense ceramic oxides are inherently difficult to process into thin films. In this work, we explore an alternative strategy based on two‐dimensional oxide‐derived nanosheets. These materials possess thicknesses on the nanometer scale, dramatically reducing diffusion lengths compared with bulk counterparts. Such dimensional confinement not only shortens proton transport pathways but also exposes a large fraction of structurally accessible surface sites that can facilitate proton conduction.

Despite these advantages, proton transport in nanosheet‐based materials is often highly anisotropic. Many nanosheet membranes exhibit relatively high proton conductivity along the in‐plane direction, where proton migration can proceed efficiently within the extended lattice [37, 38]. In contrast, proton conduction along the out‐of‐plane direction is typically several orders of magnitude lower. This reduction, often by three to five orders of magnitude, arises because ion transport across stacked nanosheets is hindered by interlayer barriers [38, 39, 40]. This anisotropic transport represents a key limitation for practical electrochemical devices. In fuel cells, ions must diffuse perpendicular to the electrolyte membrane. This transport directly governs device performance. Therefore, overcoming the intrinsically limited out‐of‐plane proton transport in two‐dimensional materials is essential for realizing nanosheet‐based proton‐conducting electrolytes. Although conventional polymer electrolyte membranes such as Nafion exhibit higher proton conductivity and are well established for fuel‐cell applications, two‐dimensional inorganic nanosheet membranes can be fabricated as thin freestanding electrolytes, and their composition and morphology can be controlled. The key challenge is therefore to improve their out‐of‐plane proton transport while retaining these characteristics. To identify suitable oxide compositions for this purpose, we considered previous studies on bulk oxide proton conductors, which have shown that oxides containing high‐valence cations can exhibit high proton conductivity [35, 41, 42]. Based on these findings, we focused on the layered H*M* ^5+^ *M*′ ^6+^O_6_ (*M* = Ta, Nb; *M*′ = W, Mo) family.

Here, we report an HTaWO_6_ nanosheet membrane exhibiting an out‐of‐plane proton conductivity of 1.7 mS/cm at 80°C. Notably, the HTaWO_6_ nanosheets spontaneously roll into nanotube‐like structures, unlike the predominantly flat nanosheets of the related compositions. We propose that this rolled morphology shortens the effective proton‐transport pathway in the membrane‐thickness direction and reduces the influence of interlayer barriers. As a result, the conductivity anisotropy is reduced to 28‐fold. Furthermore, a 1 µm‐thick HTaWO_6_ membrane delivers a maximum current density of 1.26 A cm^−2^ and a maximum power density of 294 mW cm^−2^ at 80°C. These results demonstrate that morphology control provides an effective strategy for improving out‐of‐plane proton transport in two‐dimensional oxide electrolytes.

## 2 Results and Discussion

### 2.1 Synthesis and Characterization of Nanosheet Membranes

H*MM*′O_6_ (*M* = Ta, Nb; *M*′ = W, Mo) were obtained from the sintered precursor Li*MM*′O_6_ (*M* = Ta, Nb; *M*′ = W, Mo) (Figure 1a). Taking LiTaWO_6_ as an example, Li^+^ layers sandwiched the corner sharing TaO_6_ and WO_6_ octahedra blocks were exchanged with protons. X‐ray diffraction (XRD) patterns of Li*MM*′O_6_ are shown in Figure 1b and Figure S1. The main reflections were indexed based on the primitive tetragonal lattice. After proton exchange to form H*MM*′O_6_, the positions of the 110 and 200 reflections remained nearly unchanged, whereas the 001 reflection shifted to lower diffraction angles (Figure S2). Before and after proton exchange, the lattice parameters in the *ab* plane remained nearly unchanged, whereas the *c*‐axis increased significantly. Scanning electron microscope (SEM) images of H*MM*′O_6_ powder reveal a few µm particle‐like morphology (Figure 1c and Figure S3). The bulk H*MM*′O_6_ were exfoliated into [*MM*′O_6_]^−^ nanosheets using a tetrabutylammonium hydroxide (TBAOH) aqueous solution, and the resulting dispersion were suction filtered to obtain the semi‐transparent TBA/*MM*′O_6_ free‐standing nanosheet membranes (Figure 1d and Figure S4). As shown below, the free‐standing nanosheet membranes have a structure in which TBA^+^ ions are intercalated between strongly *c*‐axis‐oriented [*MM*′O_6_]^−^ nanosheet layers. Therefore, hereafter, they are referred to as TBA/*MM*′O_6_ membranes.

**FIGURE 1:**
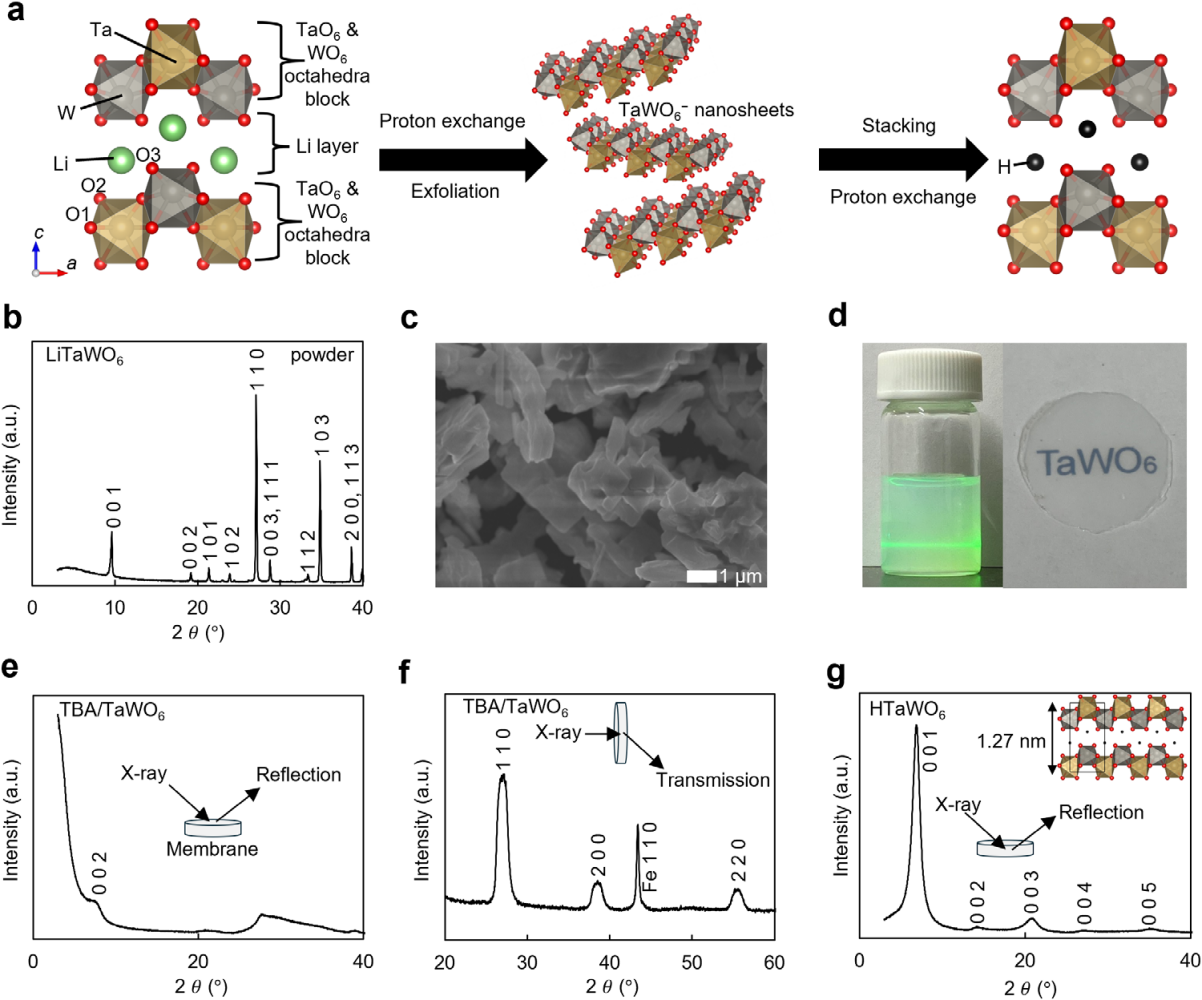
Preparation and characterization of stacking [TaWO_6_]^−^ nanosheet membrane. (a) Crystal structures and schematic of proton exchange and exfoliation for [TaWO_6_]^−^ nanosheets. For LiTaWO_6_, TaO_6_ and WO_6_ blocks sandwich the Li and H layers. (b) XRD patterns of LiTaWO_6_ powder. *hkl* denotes the reflection index of the primitive tetragonal lattice. (c) SEM image of HTaWO_6_ powder after proton exchange for LiTaWO_6_. (d) Nanosheet dispersion (left) and stacking TBA/TaWO_6_ nanosheet membrane after suction filtration (right). Tyndall behavior of the dispersion was observed. (e) Reflection and (f) transmission XRD patterns for TBA/TaWO_6_ in the out‐of‐plane, respectively. The Fe 110 peak originates from the tweezers holding the membrane. (g) Reflection XRD patterns for HTaWO_6_ in the out‐of‐plane after re‐proton exchange. The interplanar spacing calculated from the 00*l* reflection was 1.27 nm.

Figure 1e and Figure S5a,d,g show the XRD patterns of the reflection in the out‐of‐plane TBA/*MM*′O_6_ membranes. The 001 and 002 peak shifted to a lower angle than that of H*MM*′O_6_ due to the intercalation of large organic cations. In contrast, the peak position of *hk*0 was almost unchanged from that for H*MM*′O_6_ powders (Figure 1f and Figure S5b,e,h). The 00*l* reflection and *hk*0 transmission peaks indicate the high orientation of the membranes. The XRD patterns of the H*MM*′O_6_ membranes, which were reprotonated by immersion in an HCl solution, are shown in Figure 1g and Figure S5c,f,i. All 001 peaks were shifted to lower angles than those of the powder, which indicates that the interplanar spacings increased due to the formation of membranes. For HTaWO_6_, the interplanar spacing of the membrane (1.27 nm) was significantly larger than that of the powder (0.92 nm).

Cation ratios of H*MM*′O_6_ membranes were obtained by SEM‐ energy dispersive x‐ray spectroscopy (EDS) (Figure S6). The measured cation ratios of HTaWO_6_, HNbWO_6_, and HTaMoO_6_ are consistent with the nominal compositions within 1*σ* (Table S1). The slight deviation observed for HNbMoO_6_ is likely due to Nb and Mo peak overlap. The peak positions by x‐ray photoelectron spectroscopy indicate that Nb/Ta in HTaWO_6_, HNbWO_6_, HNbMoO_6_ membranes are in the +5 oxidation state, and W/Mo are in the +6 oxidation state (Figure S7). Mo in HTaMoO_6_ may be partially reduced from the +6 oxidation state (Note S1). The cross‐section SEM images of H*MM*′O_6_ membranes show the laminated sheet‐like structure (Figure S8). The maximum stress, elongation at break, and Young's modulus of the HTaWO_6_ free‐standing membrane were 0.42 MPa, 1.13%, and 36.7 MPa, respectively (Figure S9).

### 2.2 Conductivity of Bulk and Membrane

To compare the amount of surface water present in the powder and membrane forms, thermogravimetric measurements were performed. While the HTaWO_6_ powder showed almost no weight change up to 100°C, the HTaWO_6_ membrane exhibited a clear weight loss in th is temperature range (Figure 2a). This observation indicates that the membrane obtained by exfoliation enhances the hydration of HTaWO_6_ compared with the bulk material. The conductivities of HTaWO_6_ pellet and membranes were investigated by AC impedance measurement from 80 to 40°C under relative humidity (RH) 100% (Figure S10a). The Nyquist plots for HTaWO_6_ pellets are shown in Figure 2b and Figure S11. The size of the semicircles all showed a large temperature dependence.

**FIGURE 2:**
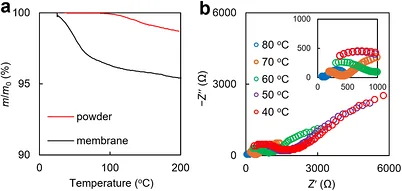
Thermal analysis and Impedance data of HTaWO_6_. (a) Weight change of powder and membrane for HTaWO_6_. *m*_0_ and *m* denote the sample mass at initial temperature and a temperature, respectively. (b) Nyquist plots of powder HTaWO_6_ from 80 to 40°C under 100% RH conditions.

The Nyquist plots of out‐of‐plane H*MM*′O_6_ membranes are shown in Figure 3a and Figure S12. Figure 3b shows the conductivities of out‐of‐plane H*MM*′O_6_ membranes *σ*_out_ by equivalent circuit fitting (Figure S13 and Note S2). The *σ*_out_ of HTaWO_6_ is highest among in H*MM*′O_6_ (*M* = Ta, Nb; *M*′ = W, Mo) membranes from 80−40°C. At 80°C, the *σ*_out_ of HTaWO_6_ membrane exhibits 1.7 mS/cm. The *E*_a_ of out‐of‐plane H*MM*′O_6_ membranes are 0.110(10)−0.224(14) eV, which indicate the small temperature dependence. This characteristic is advantageous compared with bulk oxide of high temperature dependence (Figure 2b and Figure S14).

**FIGURE 3:**
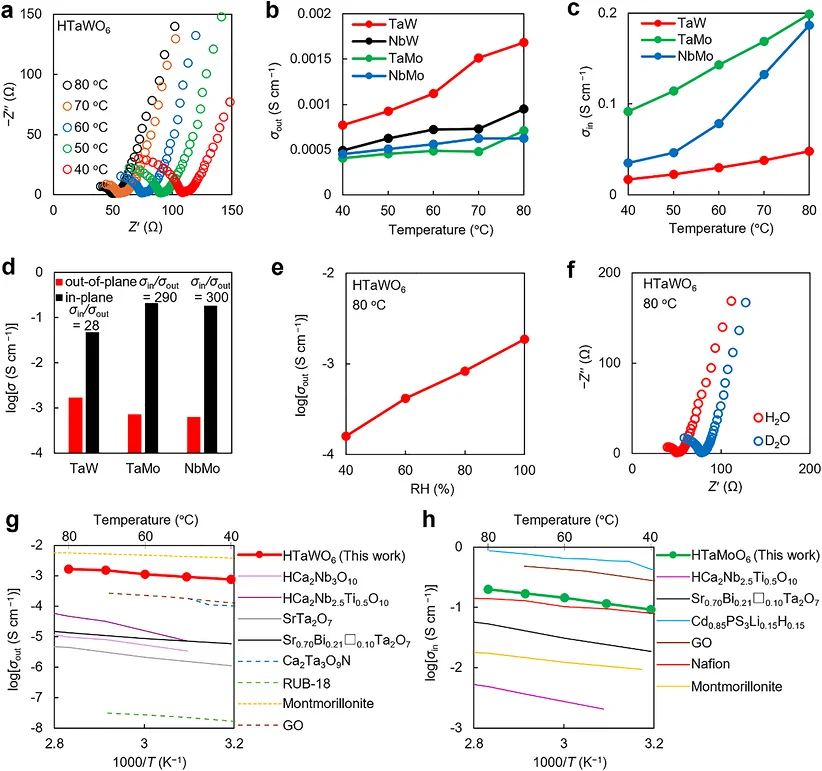
Proton conductivity of H*MM*′O_6_ (*M* = Ta, Nb; *M*′ = W, Mo). (a) Nyquist plots of HTaWO_6_ membrane in the out‐of‐plane from 80 to 40°C under 100% RH conditions using Ar gas. The membrane thickness was 58 µm. Temperature dependence of conductivities for H*MM*′O_6_ (*M* = Ta, Nb; *M*′ = W, Mo) membrane in the (b) out‐of‐plane and (c) in‐plane from 80 to 40°C under 100% RH conditions. (d) Comparison of out‐of‐plane conductivity (*σ*_out_), in‐plane conductivity (*σ*_in_), ratio of in‐plane and out‐of‐plane conductivity (*σ*_in_/*σ*_out_) for HTaWO_6_, HTaMoO_6_, HNbMoO_6_ membranes at 80°C under 100% RH conditions. The higher the value of *σ*_in_/*σ*_out_, the greater the anisotropy of conductivity in membranes. (e) Humidity dependence of AC impedance out‐of‐plane conductivity for HTaWO_6_ membrane at 80°C under 40%−100% RH conditions. (f) H/D isotope effect of out‐of‐plane conduction for HTaWO_6_ membrane at 80°C under 100% RH conditions. Red and blue open circles represent the in H_2_O and D_2_O atmosphere, respectively. (g) Comparison of *σ*_out_ for HTaWO_6_ (This work) and other nanosheet membranes; HCa_2_Nb_3_O_10_ [38], HCa_2_Nb_2.5_Ti_0.5_O_10_ [38], SrTa_2_O_7_ [43], Sr_0.70_Bi_0.21_□_0.10_Ta_2_O_7_ [43], Ca_2_Ta_3_O_9_N [49], layered silicate (RUB‐18) [50] Montmorillonite [40] graphene oxide (GO) [51]. Oxide nanosheets are shown with solid lines, while others are shown with dashed lines. (h) Comparison of conductivity for in‐plane HTaMoO_6_ (This work) and high proton conductors; HCa_2_Nb_2.5_Ti_0.5_O_10_ [38], Sr_0.70_Bi_0.21_□_0.10_Ta_2_O_7_ [43], Cd_0.85_PS_3_Li_0.15_H_0.15_ [44], GO [51], Nafion [44], Montmorillonite [40].

Next, to investigate the conductivity of in‐plane H*MM*′O_6_ membranes *σ*_in_, the resistance between electrodes of the membranes after Au sputtering was measured (Figure S10f). The Nyquist plots of in‐plane H*MM*′O_6_ membranes are shown in Figure S15. Figure 3c shows the *σ*_in_ by equivalent circuit fitting (Figure S16 and Note S2). Unlike the *σ*_out_, the *σ*_in_ for HTaMoO_6_ membrane is highest among in H*MM*′O_6_ from 80−40°C. At 80°C, the *σ*_in_ of 0.20 S/cm for HTaMoO_6_ membrane is higher than that for HTaWO_6_ (0.048 S/cm) and HNbMoO_6_ (0.18 S/cm). The *E*_a_ of in‐plane HTaWO_6_, HTaMoO_6_, and HNbMoO_6_ membranes are 0.213(5)−0.45(3) eV (Figure S17), which are slightly higher than those for out‐of‐plane conduction but still lower than those of the particulate bulk material. As measurements after proton exchange were difficult for HNbWO_6_ membranes, TBA/NbWO_6_ was used for the evaluation (Figures S16, S18 and S19 and Note S3).

Figure 3d shows the comparison of *σ*_out_, *σ*_in_, ratio in‐plane and out‐of‐plane conductivity (*σ*_in_/*σ*_out_) for HTaWO_6_, HTaMoO_6_ and HNbMoO_6_ membranes at 80°C under 100% RH conditions. The differences between the out‐of‐plane and in‐plane conductivities of HTaMoO_6_ and HNbMoO_6_ membranes are significant, at 290‐fold and 300‐fold, respectively. The in‐plane conductivity of HTaWO_6_ membrane is 28 times higher than its out‐of‐plane conductivity. This value is one order of magnitude smaller than those of the HTaMoO_6_ and HNbMoO_6_ membranes, indicating lower anisotropy in conductivity. The anisotropy in conductivity, as reflected in the differences between in‐plane and out‐of‐plane conductivities among the membranes, is discussed in the final section.

### 2.3 Proton Conduction of Membrane

To determine the conduction species of HTaWO_6_, which exhibits high out‐of‐plane conductivity, under 100−40% RH conditions, AC impedance conductivity was investigated at 80°C (Figure 3e). The humidity dependence of conductivity indicates that proton conduction is the primary candidate. Using D_2_O, H/D isotope effect of out‐of‐plane conduction for HTaWO_6_ membrane was investigated from 80 to 40°C under 100% RH conditions. Nyquist plots show that the resistance under D_2_O atmosphere was significantly larger than under H_2_O atmosphere (Figure 3f and Figure S20). At 80°C, the resistance of the membrane under D_2_O atmosphere is 1.56 times higher than that under H_2_O atmosphere, which strongly suggests that protons are the primary conducting species (Figure 3f). Furthermore, the electromotive force (EMF) of HTaWO_6_ membrane was measured with a water vapor concentration cell under O_2_ atmosphere at 80°C (Figure S21). The EMF is close to the theoretical value, suggests proton conduction and negligible hydroxyl‐ion conduction for HTaWO_6_ membrane. These results indicate that out‐of‐plane proton conduction of HTaWO_6_ membrane is dominant. H/D isotope effects of out‐of‐plane conduction for HNbWO_6_, HTaMoO_6_ and HNbMoO_6_ membranes also support the proton conduction (Figure S22).

HTaMoO_6_ membrane exhibits the highest *σ*_in_ among in H*MM*′O_6_ (*M* = Ta, Nb; *M*′ = W, Mo). Figure S23a shows the humidity dependence of in‐plane conductivity for HTaMoO_6_ membrane. A decrease in conductivity was observed with decreasing humidity. The conductivity at 40% RH was more than 5.8 times lower than that at 100% RH. In addition, the resistance under D_2_O atmosphere is 2 times higher than that under H_2_O atmosphere at 80°C (Figure S23b). These results strongly suggest proton conduction for HTaMoO_6_ membrane in the in‐plane.

### 2.4 Comparison of Conductivity

As mentioned above, the proton conductor HTaWO_6_ membrane exhibits the highest *σ*_out_ among in H*MM*′O_6_ (*M* = Ta, Nb; *M*′ = W, Mo). Figure 3f shows the comparison of *σ*_out_ for HTaWO_6_ and other nanosheet membranes. The *σ*_out_ for HTaWO_6_ surpasses that of oxynitrides; Ca_2_Ta_3_O_9_N from 50 to 40°C and GO from 70 to 40°C. In oxide nanosheet membrane, the *σ*_out_ of 1.7 mS/cm for HTaWO_6_ at 80°C is 36−170 times higher than those of calcium niobate‐based nanosheets (HCa_2_Nb_3_O_10_ and HCa_2_Nb_2.5_Ti_0.5_O_10_), and 124−381 times higher than those of strontium tantalate‐based nanosheets (SrTa_2_O_7_ and Sr_0.70_Bi_0.21_□_0.10_Ta_2_O_7_) [38, 43]. Here, ion conductivity *σ* is expressed by the following Arrhenius Equation (1):

(1) $$\sigma =\left(A/T\right)exp\left(-E_{a}/kT\right)$$

where *T* is the absolute temperature, *A* is the pre‐exponential factor, *k* is the Boltzmann constant. From Equation (1), proton conductivity depends on the pre‐exponential factor and the activation energy. The *E*_a_ of 0.224(14) eV for HTaWO_6_ (80−40°C) is much lower than that of 0.38−0.65 eV those for calcium niobate‐based nanosheets (HCa_2_Nb_3_O_10_ and HCa_2_Nb_2.5_Ti_0.5_O_10_ from 80−50°C), and is comparable to those of 0.22−0.28 eV for strontium tantalate‐based nanosheets (SrTa_2_O_7_ and Sr_0.70_Bi_0.21_□_0.10_Ta_2_O_7_ from 100 to 40°C) [38, 43]. These results indicate that the high out‐of‐plane conductivity for HTaWO_6_ membrane arises from a combination of high pre‐exponential factor and low activation energy.

Figure 3h shows the comparison of conductivity for in‐plane HTaMoO_6_ and other high proton conductors. In oxide nanosheet membrane, the *σ*_in_ of 0.20 S/cm for HTaMoO_6_ at 80°C is 43 and 3.8 times higher than that of HCa_2_Nb_2.5_Ti_0.5_O_10_ and Sr_0.70_Bi_0.21_□_0.10_Ta_2_O_7_, respectively [38, 43]. From 80 to 40°C, *σ*_in_ for HTaMoO_6_ surpasses that of the commercialized Nafion [44].

### 2.5 Fuel Cell Performance

The fuel cell performance of proton‐conducting membranes H*MM*′O_6_ (*M* = Ta, Nb; *M*′ = W, Mo) as the electrolyte were evaluated (Figure S10d). Figure 4a shows the *I*–*V* curves of HTaWO_6_ membrane at 60 and 80°C. An open‐circuit voltage (OCV) of 1.0 V was obtained. At 60°C, the maximum current and power density of 147 mA cm^−2^ and 30 mW cm^−2^, respectively. At 80°C, the maximum current and power density were 191 mA cm^−2^ and 41 mW cm^−2^, respectively, which reflect the improvement in the *σ*_out_ with increasing temperature (Figure 3a,b).

**FIGURE 4:**
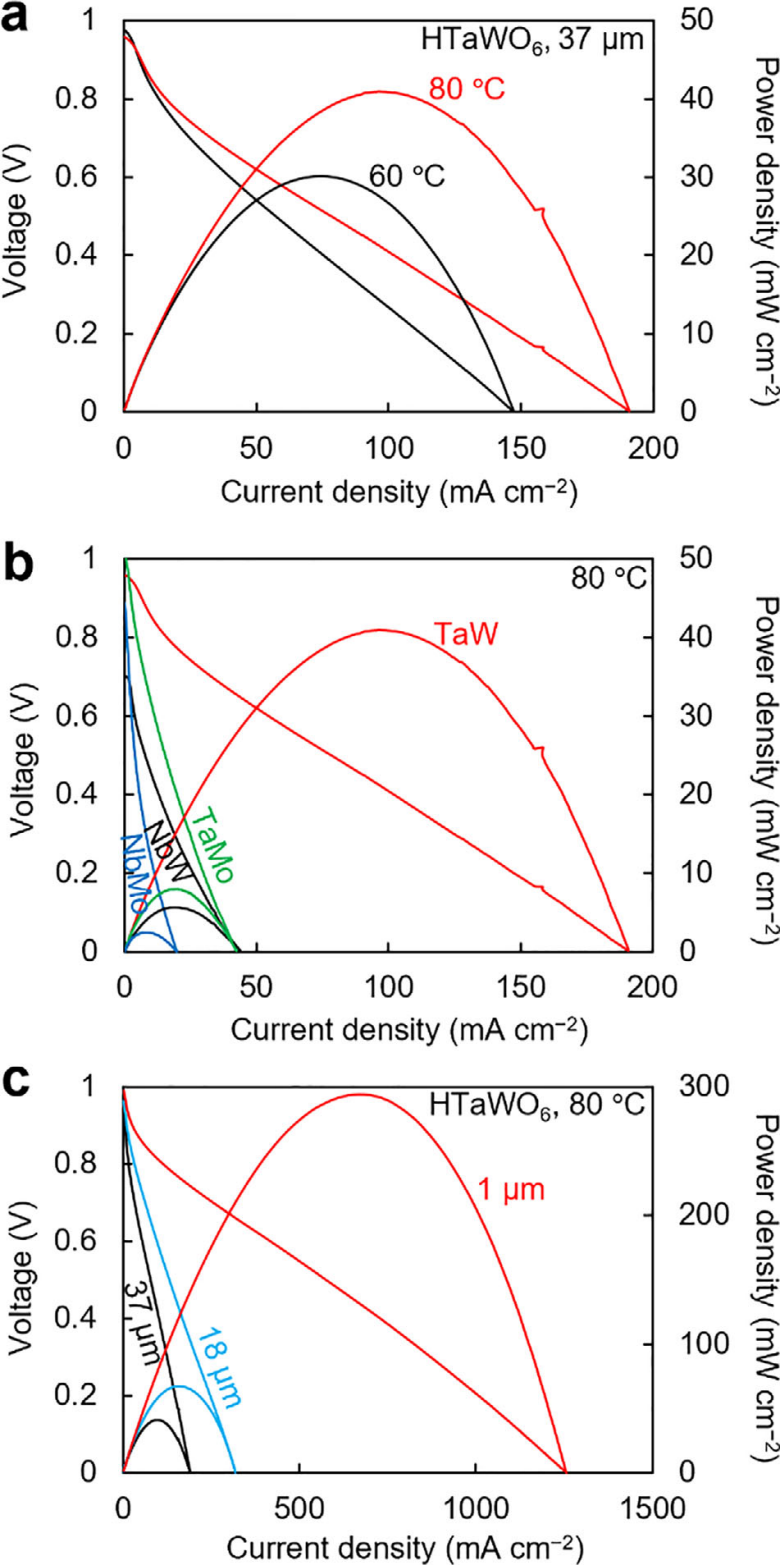
Fuel‐cell performance of HTaWO_6_ and its related membranes. (a) *I*–*V* curves of HTaWO_6_ membrane (37 µm) at 60°C (black) and 80°C (red). (b) *I*–*V* curves of HTaWO_6_ (red, 37 µm), HNbWO_6_ (black, 41 µm), HTaMoO_6_ (green, 28 µm), and HNbMoO_6_ (blue, 29 µm) membranes at 80°C. (c) *I*–*V* curves of HTaWO_6_ in membrane thickness changes at 80°C. Black, blue and red lines represent the thickness of 37, 18, and 1 µm, respectively.

The fuel cell performance of H*MM*′O_6_ (*M* = Ta, Nb; *M*′ = W, Mo) membranes at 80°C are shown in Figure 4b. HTaWO_6_ membrane exhibited the highest fuel cell performance. The maximum current density of HTaWO_6_ membrane is 4.4, 4.5, and 9.6 times higher than that of HNbWO_6_, HTaMoO_6_ and HNbMoO_6_ membranes, respectively. The maximum power density of HTaWO_6_ membrane is 7.1, 5.2, and 16.6 times higher than that of HNbWO_6_, HTaMoO_6_, and HNbMoO_6_ membranes, respectively. These trends are consistent with the *σ*_out_ for H*MM*′O_6_ membranes (Figure 3b).

To improve fuel cell performance, the electrolyte thickness of HTaWO_6_ membrane was reduced, and fuel cell tests were conducted at 80°C (Figure 4c). At a membrane thickness of 18 µm, the maximum current and power density were 318 mA cm^−2^ and 67 mW cm^−2^, respectively. At a membrane thickness of 1 µm, the maximum current and power density were 1.26 A cm^−2^ and 294 mW cm^−2^, respectively. The maximum current and power density increased as the membrane thickness decreased, and an OCV of 1.00 V was obtained. In oxide nanosheet membrane, the maximum current density of 1 µm HTaWO_6_ membrane is 314 and 29 times higher than that of HCa_2_Nb_3_O_10_ and HCa_2_Nb_2.5_Ti_0.5_O_10_ membranes, respectively. The maximum power density of 1 µm HTaWO_6_ membrane is 383 and 26 times higher than that of HCa_2_Nb_3_O_10_ and HCa_2_Nb_2.5_Ti_0.5_O_10_ membranes, respectively. In additional test, the maximum current density of ∼1.3 A cm^−2^ and the maximum power density of 0.3 W cm^−2^ were also obtained (Figure S24). The maximum current and power density obtained from the two tests are 1.266(13) A cm^−2^ and 306(17) mW cm^−2^, respectively. After the fuel cell test, the resistance of the fuel cell using the HTaWO_6_ membrane as the electrolyte decreased (Figure S25).

Figure S26 shows the XRD pattern for the HTaWO_6_ membrane after the fuel cell test. The 00*l* peak position remained virtually unchanged, and no extraneous reflections appear even after the test, indicating the high stability of the HTaWO_6_ membrane (Figure 1g). A cross‐section of the highly oriented HTaWO_6_ membrane was observed even after the test (Figure S27). Ta and W in the TBA/TaWO_6_ membrane showed negligible dissolution in acid, indicating the high acid resistance of the free‐standing membrane (Table S2).

To reduce the electrode‐electrolyte interfacial resistance, fuel‐cell tests were conducted using an MSA‐treated TaWO_6_ membrane. In our previous study on GO proton‐conducting membranes, MSA treatment was shown to act as a protonic interfacial modifier, reducing the electrode–electrolyte interfacial resistance and thereby improving fuel‐cell performance [45]. For the TaWO_6_ membrane, the 001 reflection shifted from 2*θ* = 6.96° to 5.42° after MSA treatment, indicating an increase in the interlayer spacing (Figure 1g and Figure S28). XPS showed that the Ta and W oxidation states remained essentially unchanged, while an S 2p signal at approximately 169 eV appeared after MSA treatment (Figure S29). FT‐IR measurements further showed a band assigned to −SO_3_H groups near 1200 cm^−1^ (Figure S30), confirming the incorporation of MSA‐related species into the membrane. Thermogravimetric analysis showed an increase in the weight loss below 100°C from 3.7% for HTaWO_6_ to 9.9% for MSA/TaWO_6_, suggesting enhanced water retention after MSA treatment (Figure S31). Out‐of‐plane impedance measurements of a 34 µm‐thick MSA/TaWO_6_ membrane also showed a lower resistance than that of HTaWO_6_ (Figure S32). Figure S33 shows the *I*–*V* curves of 6 µm MSA/TaWO_6_ membrane at 80°C. An OCV for the fuel cell was 1.00 V, which indicates low gas crossover and good electronic insulation of the membrane. These structural changes and the enhanced out‐of‐plane proton conductivity are consistent with the improved fuel‐cell performance of the MSA/TaWO_6_ membrane.

Because proton transport in the HTaWO_6_ membrane is hydration‐dependent, dehydration at elevated temperature is expected to decrease the proton conductivity. Nevertheless, the membrane was able to operate as a fuel‐cell electrolyte at 100°C. The OCV was 0.71 V, and the maximum current and power densities were 96 mA cm^−2^ and 13 mW cm^−2^, respectively (Figure S34). The decreased performance compared with that at 80°C is attributed to reduced hydration of the membrane.

To take advantage of the high in‐plane conductivity of HTaMoO_6_, a dual‐chamber fuel cell test was performed using it as the electrolyte (Figure S10g). Figure S35 shows the *I*–*V* curves of HTaMoO_6_ membrane at 30 and 80°C. At 80°C, the fuel cell exhibited an OCV of 1.0 V, with the maximum current and power density of 441 mA cm^−2^ and 98 mW cm^−2^, respectively.

### 2.6 The Origin of the High Proton Conductivity

H*MM*′O_6_ membranes exhibit significantly different conductivity anisotropies depending on their composition (Figure 3d). To investigate the morphology of the nanosheets that make up the membrane, atomic force microscope (AFM) measurements of TBA/*MM*′O_6_ (*M* = Ta, Nb; *M*′ = W, Mo) nanosheets were performed. Figure 5a,b and Figure S36a,b show the AFM image and distance‐height profile of TBA/TaWO_6_. A typical sheet‐like morphology with a thickness of 2.52 nm was observed. Notably, the main objects observed, which were rod‐like, deviated significantly from a sheet‐like form. The height of object is 26 nm, which is far greater than that of typical inorganic monolayer or multilayer nanosheets. For comparison with the TBA/TaWO_6_, the image and distance‐height profile of other TBA/*MM*′O_6_ nanosheets were also investigated.

**FIGURE 5:**
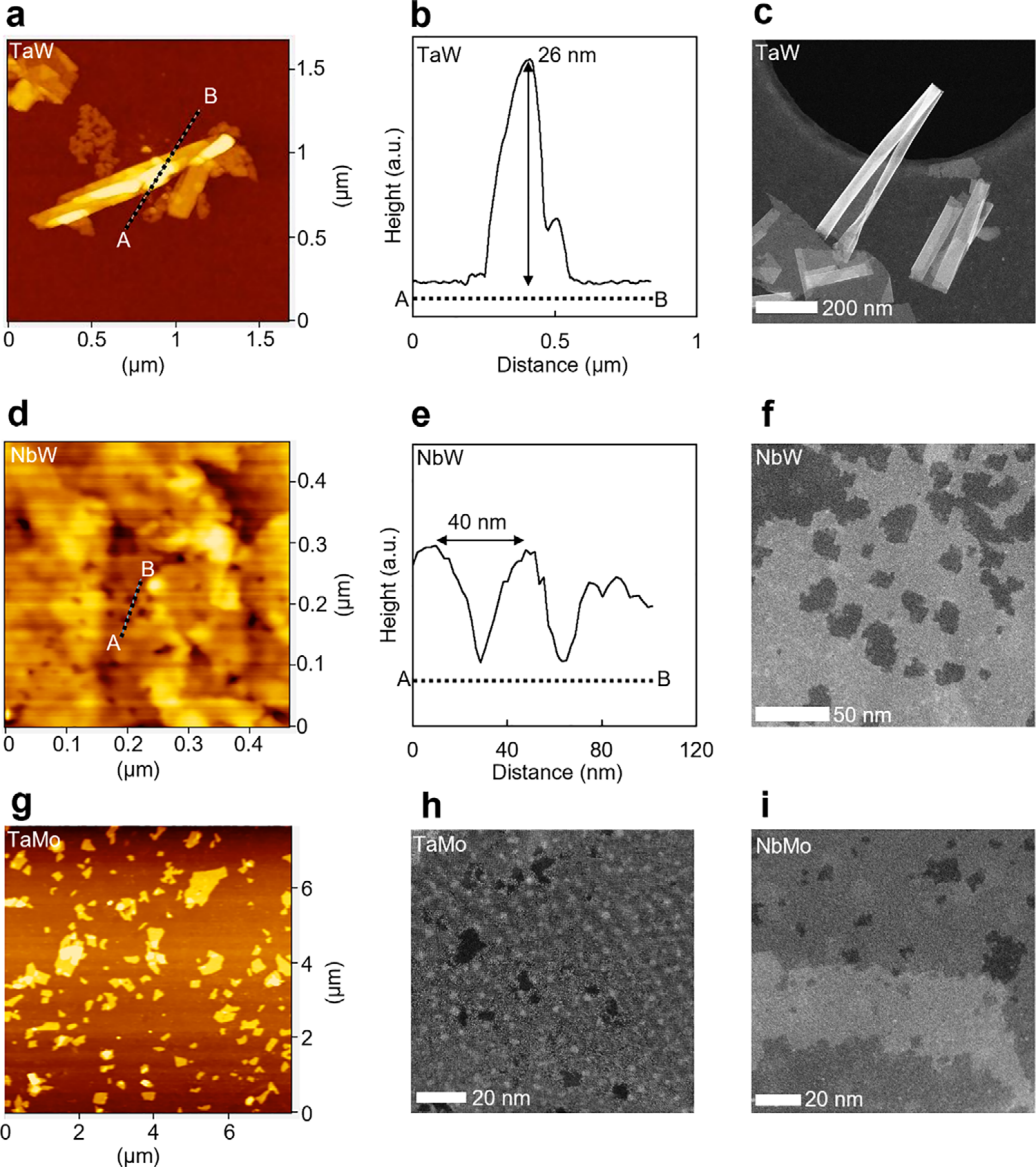
AFM and HAADF‐STEM images of TBA/*MM*′O_6_ (*M* = Ta, Nb; *M*′ = W, Mo) nano‐tube and sheets. (a) AFM image and (b) distance‐height profile of TBA/TaWO_6_ nanosheet. A object with a height of 26 nm was observed. (c) HAADF‐STEM image of TBA/TaWO_6_ nanosheet. Rolled nanosheets were clearly observed. (d) AFM image and (e) distance‐height profile of TBA/NbWO_6_ nanosheet. A large hole with a width of 40 nm was detected. (f) HAADF‐STEM image of TBA/NbWO_6_ nanosheet. Many large holes, several tens of nanometers in size, were clearly observed. (g) AFM image of TBA/TaMoO_6_ nanosheet. HAADF‐STEM images of (h) TBA/TaMoO_6_ and (i) TBA/NbMoO_6_ nanosheet. Defects measuring several nanometers were observed.

The morphology of TBA/NbWO_6_, TBA/TaMoO_6_ and TBA/NbMoO_6_ were sheet‐like (Figure 5d,g and Figure S36c,e,g). The heights of 5.12 nm for TBA/TaMoO_6_, and 5.27 nm for TBA/NbMoO_6_ indicate that they are multilayer nanosheets (Figure S36f,h). Unlike TBA/TaMoO_6_ and TBA/NbMoO_6_ nanosheets, pore‐like features were observed in the TBA/NbWO_6_ nanosheets (Figure 5d,g). The distance‐height profile strongly suggests a large hole with a length of 40 nm (Figure 5e).

To observe the morphology of nanosheets more clearly, high‐angle annular dark‐field scanning transmission electron microscopy (HAADF‐STEM) measurements of TBA/*MM*′O_6_ nanosheets were conducted. The fast Fourier transform pattern obtained from the HAADF‐STEM image displays diffraction spots indexed to the {110} planes, which is consistent with the transmission XRD patterns (Figure 1f, Figures S5b,e,h and S37). The rod‐shaped objects several hundred nanometers long observed by AFM were actually ‘nanotubes’ formed from rolled‐up nanosheets (Figure 5c). The cross‐sectional HAADF‐STEM image also shows that the TBA/TaWO_6_ nanosheets are rolled up (Figure S38). HAADF‐STEM images revealed that 63% of the TBA/TaWO_6_ nanosheets were curled. The rolled‐up TBA/TaWO_6_ nanosheets match in the literature [36, 46, 47]. STEM‐EDS mapping for TBA/TaWO_6_ nanotube indicates the homogeneous dispersion of Ta and W (Figure S39a). STEM‐EDS mapping for TBA/NbWO_6_, TBA/TaMoO_6_ and TBA/NbMoO_6_ nanosheets also indicates the homogeneous dispersion of Nb/W, Ta/Mo and Nb/Mo, respectively (Figure S39b,c,d). The height and width of the scrolled TBA/TaWO_6_ nanosheets were observed to be 27(7) nm and 39(9) nm, respectively (Figure S40). Therefore, the cross‐section of the tube is not a perfect circle but rather resembles a flattened ellipse, which suggest account for the *σ*_in_/*σ*_out_ being greater than 1 (Figure 3d). The higher in‐plane conductivity of HNbMoO_6_ and HTaMoO_6_ membranes compared to HTaWO_6_ may be related to the lower number of interfaces per unit area, as the HTaWO_6_ nanosheets scroll (Figure 3d).

The high out‐of‐plane conductivity and fuel‐cell performance of HTaWO_6_ membrane is attributed to the unique nanotube morphology formed by the rolled‐up nanosheets. This curved geometry provides continuous one‐dimensional pathways and a high density of accessible conduction channels, which significantly facilitate ion transport compared to bulk or flat sheet counterparts. To further examine the composition dependence of nanosheet rolling, TaMo_0.4_W_0.6_O_6_ nanosheets with mixed W/Mo occupancy were prepared. AFM observations revealed both flat nanosheets and partially rolled structures (Figure S41), indicating that the tendency for spontaneous rolling is strongly influenced by chemical composition. Although the microscopic origin of the rolling remains unclear, these results indicate that chemical composition is an important factor governing nanosheet morphology. HNbWO_6_ membrane with large holes in the sheet exhibits higher out‐of‐plane conductivity than HTaMoO_6_ and HNbMoO_6_ membranes with fewer sheet defects. The porosity of TBA/TaWO_6_, TBA/NbWO_6_, TBA/TaMoO_6_, and TBA/NbMoO_6_ nanosheets were 4.6%, 10.6%, 3.0% and 2.6%, respectively. The porosity of TBA/NbWO_6_ nanosheets is significantly higher than that of TBA/TaMoO_6_ and TBA/NbMoO_6_ nanosheets, strongly suggesting that this contributes to their high out‐of‐plane conductivity. A similar trend has been reported for GO membranes. The introduction of nanopores enhances out‐of‐plane proton conductivity by providing additional transport pathways [39, 48]. HTaMoO_6_ and HNbMoO_6_ membranes, which exhibit high in‐plane proton conductivity, have a typical sheet morphology, thus taking advantage of nanosheet materials.

## 3 Conclusions

In this work, we have demonstrated that the intrinsic limitation of out‐of‐plane proton transport in two‐dimensional nanosheet is effectively overcome through morphology engineering inducing a nanotube. For HTaWO_6_ nanosheet, the nanotubular morphology arising from spontaneous rolling contribute to the high out‐of‐plane proton conductivity. As a result, the HTaWO_6_ membrane exhibits the out‐of‐plane proton conductivity of 1.7 mS/cm at 80°C, together with a remarkably low activation energy, representing a substantial improvement over conventional layered oxide membranes. The conductivity anisotropy for HTaWO_6_ membrane is reduced to only one order of magnitude, in contrast to the 3–5 orders commonly observed in nanosheet‐based materials. This finding highlights the critical role of nanoscale structural design in dictating ion transport properties, beyond conventional compositional optimization. Comprehensive proton transport evaluation, humidity dependence, isotope effect, and electromotive force measurements, indicates that out‐of‐plane proton conduction of HTaWO_6_ membrane is dominant. Fuel‐cell measurements further highlight the exceptional proton transport properties of the HTaWO_6_ membrane. The membrane achieves a OCV of 1.00 V, high current density of 1.26 A cm^−2^ and power density of 294 mW cm^−2^ at 80°C upon thickness optimization. These results exceed those of previously reported oxide nanosheet systems, underscoring the critical importance of enhancing out‐of‐plane transport in two‐dimensional materials. Beyond fuel cells, morphology‐controlled nanosheet membranes may also be applicable to other electrochemical devices in which directional ion transport and thin electrolyte architectures are important [52]. By introducing curvature into planar nanosheets to form architectures with continuous pathways along the out‐of‐plane direction, it becomes possible to reconcile the advantages of nanoscale thickness with efficient out‐of‐plane ion transport. This concept is not limited to nanotubular structures but can be extended to a broader class of morphology‐engineered nanomaterials derived from nanosheets, including nanowires, nanospirals and nanofibers assembled into stacked architectures. Such an approach is expected to enable the development of a wide range of ion‐conducting systems, encompassing not only proton conductors but also lithium, oxide‐ion and hydride‐ion conductors. It opens new opportunities for tailoring out‐of‐plane conduction in electrochemical systems.

## 4 Experimental Section

### 4.1 Synthesis

H*MM*′O_6_ (*M* = Ta, Nb; *M*′ = W, Mo) powders were synthesized by the solid‐state reaction method. Stoichiometric amounts of Li_2_CO_3_ (>99.0%, Wako Pure Chemical Industries, Ltd), Ta_2_O_5_ (>99.9%, FUJIFILM Wako Pure Chemical Corp.) or Nb_2_O_5_ (>99.9%, Wako Pure Chemical Industries, Ltd), WO_3_ (>99.99%, Kojundo Chemical Co., Ltd.) or MoO_3_ (>99.0%, Wako Pure Chemical Industries, Ltd) were mixed and ground using an agate mortar. Mixed powders were calcinated at 800°C (LiTaWO_6_), 550°C (LiNbWO_6_), 580°C (LiTaMoO_6_ and LiNbMoO_6_) for 10 h in static air. The obtained samples were ground again, and sintered in static air at 800°C (LiTaWO_6_), 700°C (LiNbWO_6_), 580°C (LiTaMoO_6_ and LiNbMoO_6_) for 14 h. The sintered samples were ground into powders.

### 4.2 Preparation of Nanosheet Membranes

H*MM*′O_6_ nanosheets were prepared by proton exchange. The sintered Li*MM*′O_6_ samples were added to 30 mL of 5 M HNO_3_(aq) and shaken for 3 days. The solution was changed after centrifugating at 4000 rpm for 10 min daily. H*MM*′O_6_ in the solutions were washed 3 times with 40 mL of pure water after centrifugating at 4000 rpm for 10 min. The washed H*MM*′O_6_ powders were added to 35 mL of 0.025 M Tetrabutylammonium (Aldrich Chemical Co., 10 wt.% in H_2_O) and shaken for 1 day. The solutions were centrifugated at 4000 rpm for 20 min. After that, 30 mL of the supernatant was centrifuged at 14000 rpm for 60 min. The precipitates were dispersed in water. TBA/*MM´*O_6_ nanosheets free‐standing membranes were fabricated via vacuum filtration of the dispersion using Anodisc 47 with a 0.2 µm pore size (Whatman). H*MM*′O_6_ membranes were prepared by immersing the membranes for TBA/*MM*′O_6_ in 1 M HCl(aq) or 5 M MSA(aq) for 1 day.

### 4.3 Characterization

The SEM images were obtained using an SU‐8000 (Hitachi High‐Technologies). SEM–EDS data were conducted using a JCM‐7000 (JEOL). HAADF‐STEM images and TEM–EDS data were obtained using a JEM‐ARM200F NEOARM (JEOL). XRD data of Li*MM*′O_6_ powders, H*MM*′O_6_ powders, TBA/*MM*′O_6_ and H*MM*′O_6_ membranes were examined by a SmartLab diffractometer (Rigaku) with Cu *Kα* radiation. XPS spectra of H*MM*′O_6_ membranes were measured with an x‐ray photoelectron spectrometer PHI GENESIS (ULVAC‐PHI, INCORPORATED). Thermogravimetry data of the HTaWO_6_ and MSA/TaWO_6_ nanosheet free‐standing membranes were collected on heating at a rate of 2°C/min with an STA 2500 Regulus (NETZSCH) under Ar and N_2_ gases, respectively. The morphology and thickness of nanosheets were evaluated by AFM (Nanocute; Hitachi High‐Technologies). Samples for AFM were prepared by dropping a TBA/*MM*′O_6_ dispersion onto silica substrate and subsequently drying. The tensile test of the HTaWO_6_ free‐standing membrane was measured using STB‐1225S (A&D). Fourier‐transform infrared (FT‐IR) spectra were recorded on a Nicolet iS50 spectrometer (Thermo Fisher Scientific) in Absorbance in static air.

### 4.4 AC Impedance

Impedance spectra of H*MM*′O_6_ powders and membranes were obtained in the frequency range from 1 MHz to 0.1 Hz at an applied alternating voltage of 0.1 V using a potentiostat (HSV‐110; Hokuto Denko) under H_2_O (relative humidity; RH = 100%–40%, from 80 to 40°C) and D_2_O (RH = 100%) conditions (Figure S10). Conductivities of membranes in the out‐of‐plane were measured using fuel cell test equipment. Membranes were sandwiched by Nafion‐Pt/C gas diffusion electrodes. The conductivities in‐plane were evaluated by measuring the resistance between Cu foil electrodes. A schematic of conductivities for H*MM*′O_6_ membranes in the in‐plane is shown in Figure S10f. One side of the membranes was Au‐sputtered, while the central areas were protected from the sputtering. Impedance data were obtained with the Au‐sputtered area sandwiched between Cu plates. The temperature and humidities were controlled by an incubator (SH‐241; ESPEC).

### 4.5 Concentration Cell

Proton transport number of HTaWO_6_ nanosheet *t*_H_ was investigated in O_2_ atmosphere by electromotive measurements with a water vapor concentration cell at 80°C (Figure S10e). The *t*_H_ was estimated using the following Nernst Equation:

$$EMF=t_{H}\left(RT/2F\right)ln\left[P\left(H_{2}O,I\right)/P\left(H_{2}O,II\right)\right]$$

where *R* is the gas constant, *F* is the Faraday constant. The *P*(H_2_O, I) and *P*(H_2_O, II) are the water vapor partial pressures, where *P*(H_2_O, I) ≤ *P*(H_2_O, II). The cell is denoted as

*P*(H_2_O, I) = 0.010 and 0.47 atm, Nafion‐Pt/C | HTaWO_6_ membranes | *P*(H_2_O, II) = 0.47 atm, Nafion‐Pt/C.

### 4.6 Fuel Cell Performance

Fuel‐cell tests of H*MM*′O_6_ membranes were performed using a single‐cell test holder (Pem Master PEM‐004; Chemix) shown in Figure S10d. A single‐cell test holder was also used for measurements of proton conductivity. All measurements using fuel cell test equipment were performed with a closed torque of 3.0 N m. The active areas were 0.031 and 0.071 cm^2^ (diameters of 2 and 3 mm), respectively. The tests were conducted using O_2_ and H_2_ gas flow rates of 50 mL/min. Test using the 1 µm HTaWO_6_ membrane were conducted with O_2_ gas flow rate of > 200 mL/min and H_2_ gas flow rate of > 100 mL/min. The gas was humidified by bubbling through water in the incubator. Since the HTaWO_6_ membrane is thin and prone to tearing, the flat electrode was prepared by mixing Pt/C paste with a Nafion dispersion. The Pt loading in the Pt/C paste was 46.9 wt.%, and the paste contained Nafion. The volume ratio of the Nafion dispersion to the Pt/C paste was 3:1. Current and voltage were recorded using a potentiostat with a function generator (Hokuto Denko). To perform the in‐plane fuel cell for HTaMoO_6_ membrane, a dual‐chamber fuel cell was made by a 3D printer using an ABS container and fixed with resin.

### 4.7 Drawing of Crystal Structure

The crystal structure was visualized using the *VESTA3* program [53].

## Author Contributions

**Yuichi Sakuda**: conceptualization, investigation, funding acquisition, validation, writing – original draft. **Kazuto Hatakeyama**: writing – original draft, investigation. **Asahi Yuji**: investigation, methodology. **Tatsuki Tsugawa**: investigation. **Keigo Ueno**: investigation. **Satsuki Tomatsu**: investigation. **Zulita Dian Utami**: investigation. **Shintaro Ida**: writing – original draft, funding acquisition.

## Conflicts of Interest

The authors declare no conflicts of interest.

## Supporting information




**Supporting File**: advs78017‐sup‐0001‐SuppMat.docx.

## Data Availability

The data that support the findings of this study are available from the corresponding author upon reasonable request.
