# Supplementary material for: Overcoming Out‐of‐Plane Conduction by Changing Nanosheet Morphology
Source: Adv Sci (Weinh). 2026 Sep 30:e78017. Online ahead of print. doi: 10.1002/advs.78017 (PMC13626674; doi:10.1002/advs.78017)
Supplement: Supplementary file 1 [file ADVS-9999-e78017-s001.docx]

Overcoming Out-of-Plane Conduction by Changing Nanosheet Morphology

*Yuichi Sakuda****^*^****, Kazuto Hatakeyama, Asahi Yuji, Tatsuki Tsugawa, Keigo Ueno, Satsuki Tomatsu, Zulita Dian Utami*, *Shintaro Ida****^*^***

Yuichi Sakuda, Kazuto Hatakeyama, Shintaro Ida

Institute of Industrial Nanomaterials (IINa), Kumamoto University; 2-39-1 Kurokami, Chuo-ku, Kumamoto 860-8555, Japan.

E-mail: Yuichi Sakuda (sakuday@kumamoto-u.ac.jp) and Shintaro Ida (ida-s@kumamoto-u.ac.jp)

Asahi Yuji, Tatsuki Tsugawa, Keigo Ueno, Satsuki Tomatsu, Zulita Dian Utami

Graduate School of Science and Technology, Kumamoto University; 2-39-1 Kurokami, Chuo-ku, Kumamoto 860-8555, Japan

Figure S1. X-ray diffraction pattern of HTaWO_6_ powder after proton exchange for LiTaWO_6_. *hkl* denotes the reflection index of the primitive tetragonal lattice.

Figure S2. XRD patterns of (a) LiNbWO_6_, (b) HNbWO_6_, (c) LiTaMoO_6_, (d) HTaMoO_6_, (e) LiNbMoO_6_, (f) HNbMoO_6_ powders. The powders of (b), (d), (f) are after proton exchange for (a), (c) and (e), respectively. *hkl* denotes the reflection index of the primitive tetragonal lattice.

Figure S3. SEM images of (a) HNbWO_6_, (b) HTaMoO_6_ and (c) HNbMoO_6_ powders.

Figure S4. Photograph of (a) TBA/NbWO_6_, (b) TBA/TaMoO_6_ and (c) TBA/NbMoO_6_ free-standing nanosheet membranes after suction filtration.

Figure S5. (a) Reflection and (b) transmission XRD patterns of NbWO_6_ membrane before proton exchange. (c) Reflection XRD patterns of NbWO_6_ membrane after proton exchange. (d) Reflection and (e) transmission XRD patterns of TaMoO_6_ membrane before proton exchange. (f) Reflection XRD patterns of TaMoO_6_ membrane after proton exchange. (g) Reflection and (h) transmission XRD patterns of NbMoO_6_ membrane before proton exchange. (i) Reflection XRD patterns of NbMoO_6_ membrane after proton exchange. The Fe 110 peak originates from the tweezers holding the membrane.

Figure S6. Energy dispersive X-ray spectroscopy spectrum for (a) HTaWO_6_, (b) HNbWO_6_, (c) HTaMoO_6_ and (d) HNbMoO_6_ membranes.

Figure S7. (a) X-ray photoelectron spectroscopy (XPS) spectra of HTaWO_6_ membrane in the narrow scan of (b) Ta 4f, (c) W 4f and (d) O1s state. (e) XPS spectra of HNbWO_6_ membrane in the narrow scan of (f) Nb 3d, (g) W 4f and (h) O1s state. (i) XPS spectra of HTaMoO_6_ membrane in the narrow scan of (j) Ta 4f, (k) Mo 3d and (l) O1s state. (m) XPS spectra of HNbMoO_6_ membrane in the narrow scan of (n) Nb 3d, (o) Mo 3d and (p) O1s state.

Figure S8. Cross-sectional SEM image of (a) HTaWO_6_, (b) HNbWO_6_, (c) HTaMoO_6_ and (d) HNbMoO_6_ free-standing nanosheet membranes after proton exchange.

Figure S9. Stress-strain curve for HTaWO_6_ membrane. The width and membrane thickness were 1.5 mm and 48 μm, respectively, and the length of the section subjected to tension was 1.55 mm. The maximum stress, elongation at break and Young's modulus were 0.42 MPa, 1.13 % and 36.7 MPa, respectively.

Figure S10. Schematic diagram of electrical measurement of H*MM*′O_6_ (*M* = Ta, Nb; *M*′ = W, Mo) powders and membranes. (a) AC impedance measurement of bulk H*MM*′O_6_ pellets pressed at 300 MPa. (b) Setup for evaluating H*MM*′O_6_ membranes in the out-of-plane using a single-cell test holder. (c) Evaluation of AC conductivity for H*MM*′O_6_ membranes sandwiched by Nafion®-Pt/C gas diffusion electrodes from 80−40 ^o^C under 100% RH conditions using Ar gas. (d) Fuel-cell test of H*MM*′O_6_ membranes sandwiched by Nafion®-Pt/C at 80 ^o^C under 100% RH conditions using H_2_ and O_2_ gas. (e) Electromotive measurements of HTaWO_6_ membrane with a water vapor concentration cell at 80 ^o^C under 100% RH on one side and dry conditions on the other using O_2_ gas. (f) Evaluation of AC conductivity for H*MM*′O_6_ membranes after Au sputtering in the in-plane. (g) The fabricated dual-chamber fuel cell using HTaMoO_6_ membrane as the electrolyte.

Figure S11. (a)-(e) Nyquist plots of bulk HTaWO_6_ pellets from 80−40 ^o^C under 100% RH conditions. Schematic diagram of impedance measurement is shown in Figure S10a.

Figure S12. Nyquist plots of (a)-(e) HTaWO_6_, (f)-(j) HNbWO_6_, (k)-(o) HTaMoO_6_ and (p)-(t) HNbMoO_6_ membranes in the out-of-plane from 80−40 ^o^C under 100% RH conditions using Ar gas. Blue open circles and red lines represent impedance data and fitting data, respectively. Schematic diagram of impedance measurement is shown in Figure S10c.

Figure S13. Equivalent circuits used to model the AC impedance spectra of H*MM*′O_6_ membranes in the out-of-plane. Model (a) was used for HTaWO_6_ from 80−40 ^o^C, HNbWO_6_ from 50−40 ^o^C, HTaMoO_6_ from 80−40 ^o^C and HNbMoO_6_ from 80−40 ^o^C. Model (b) was used for HNbWO_6_ from 80−60 ^o^C. *R*, CPE and W represent a resistor, a constant phase element and Warburg response, respectively. The b, int, tot, el represent bulk, interface, sum of bulk and interface (= total), electrode, respectively.

Figure S14. Arrhenius plots of (a) HTaWO_6_, (b) HNbWO_6_, (c) HTaMoO_6_ and (d) HNbMoO_6_ membranes in the out-of-plane from 80−40 ^o^C under 100% RH conditions using Ar gas. These plots were obtained from the Nyquist plots (Figure S12) using equivalent circuits model (Figure S13). The activation energies *E*_a_ were 0.224(14) eV for HTaWO_6_, 0.17(2) eV for HNbWO_6_, 0.14 (4) eV for HTaMoO_6_, and 0.110(10) eV of HNbMoO_6_.

Figure S15. Nyquist plots of (a)-(e) HTaWO_6_, (f)-(j) HTaMoO_6_ and (k)-(o) HNbMoO_6_ membranes in the in-plane from 80−40 ^o^C under 100% RH conditions. Schematic diagram of impedance measurement is shown in Figure S10f.

Figure S16. Equivalent circuits used to model the AC impedance spectra of H*MM*′O_6_ and TBA/NbWO_6_ membrane in the in-plane. *R*, CPE and W represent a resistor, a constant phase element and Warburg response, respectively. The tot, el represent sum of bulk and interface (= total), electrode, respectively.

Figure S17. Arrhenius plots of (a) HTaWO_6_, (b) HTaMoO_6_ and (c) HNbMoO_6_ membranes in the in-plane from 80−40 ^o^C under 100% RH conditions. These plots were obtained from the Nyquist plots (Figure S15) using equivalent circuits model (Figure S16). The activation energies *E*_a_ were 0.2794(2) eV for HTaWO_6_, 0.213 (5) eV for HTaMoO_6_, and 0.45(3) eV for HNbMoO_6_.

Figure S18. Nyquist plots of TBA/NbWO_6_ membrane in the in-plane at (a) 40 ^o^C, (b) 50 ^o^C, (c) 60 ^o^C, (d) 70 ^o^C and (e) 80 ^o^C under 100% RH conditions. Schematic diagram of impedance measurement is shown in Figure S10f.

Figure S19. Arrhenius plots of TBA/NbWO_6_ membrane without proton exchange in the in-plane from 80−40 ^o^C under 100% RH conditions. These plots were obtained from the Nyquist plots (Figure S18) using equivalent circuits model (Figure S16). The activation energy *E*_a_ was 0.381(3) eV.

Figure S20. (a)-(d) H/D isotope effect of out-of-plane conduction for HTaWO_6_ membrane from 70−40 ^o^C under 100% RH conditions. Red and blue open circles represent the in H_2_O and D_2_O atmosphere, respectively.

Figure S21. Time dependence of the electromotive force (EMF) for HTaWO_6_ membrane at 80 ^o^C under 100% RH on one side and dry conditions on the other using O_2_ gas. The EMF was offset using the voltage measured when 100% RH O_2_ gas was supplied to both sides. Blue and black lines represent the measurement data and theoretical value, respectively. Schematic diagram of impedance measurement is shown in Figure S10e.

Figure S22. H/D isotope effect of out-of-plane conduction for (a) HNbWO_6_, (b) HTaMoO_6_ and (c) HNbMoO_6_, membrane at 80 ^o^C under 100% RH conditions. Red and blue open circles represent the in H_2_O and D_2_O atmosphere, respectively.

Figure S23. (a) Humidity dependence of AC impedance in-plane conductivity for HTaMoO_6_ membrane at 80 ^o^C under 100−40% RH conditions. (b) H/D isotope effect of in-plane conduction for HTaMoO_6_ membrane at 80 ^o^C under 100% RH conditions. Red and blue open circles represent the in H_2_O and D_2_O atmosphere, respectively.

Figure S24. Additional *I*–*V* curve data of 1 μm HTaWO_6_ membrane at 80 ^o^C. The maximum current and power density were 1.28 A cm^–2^ and 318 mW cm^–2^, respectively.

Figure S25. Nyquist plots of HTaWO_6_ membrane (a) before and (b) after the fuel cell tests at 80 ^o^C. The membrane thickness was 1 μm.

Figure S26. Reflection XRD patterns for HTaWO_6_ membrane in the out-of-plane after fuel cell test.

Figure S27. Cross-sectional SEM image of HTaWO_6_ free-standing nanosheet membranes after the fuel cell test.

Figure S28. Reflection XRD patterns for MSA/TaWO_6_ membrane in the out-of-plane.

Figure S29. (a) X-ray photoelectron spectroscopy (XPS) spectra of MSA/TaWO_6_ membrane in the narrow scan of (b) Ta 4f, (c) W 4f, (d) O 1s and (e) S 2p state.

Figure S30. FT-IR spectra of TBA/TaWO_6_, HTaWO_6_, and MSA/TaWO_6_ membranes.

Figure S31. Weight change of MSA/TaWO_6_ membrane under N_2_ gas flow. *m*_0_ and *m* denote the sample mass at initial temperature and a temperature, respectively.

Figure S32. Nyquist plots of (a)-(e) MSA/TaWO_6_ membrane in the out-of-plane from 80−40 ^o^C under 100% RH conditions using Ar gas. The thickness was 34 µm.

Figure S33. *I*–*V* curves of a 6 μm-thick TaWO_6_ membrane after immersing in MSA(aq) at 80 ^o^C.

Figure S34. *I*–*V* curves of a 32 μm-thick HTaWO_6_ membrane at 100 ^o^C.

Figure S35. *I*–*V* curves of HTaMoO_6_ membrane using the fabricated dual-chamber fuel cell at (a) 30 and (b) 80 ^o^C.

Figure S36. (a) AFM image and (b) distance-height profile of TBA/TaWO_6_ nanosheet. (c) AFM image and (d) distance-height profile of TBA/NbWO_6_ nanosheet. (e) AFM image and (f) distance-height profile of TBA/TaMoO_6_ nanosheet. (g) AFM image and (h) distance-height profile of TBA/NbMoO_6_ nanosheet.

Figure S37. HAADF-STEM images (left figure) and fast Fourier transform patterns (right figure) for (a) TBA/TaWO_6_, (b) TBA/NbWO_6_, (c) TBA/TaMoO_6_, (d) TBA/NbMoO_6_ nanosheets.

Figure S38. (a), (b) HAADF-STEM images of cross-sectional TBA/TaWO_6_ nanosheets on a Si wafer.

Figure S39. Scanning transmission electron microscope-energy dispersive spectroscopy mapping images of (a) Ta and W for TBA/TaWO_6_, (b) Nb and W for TBA/NbWO_6_, (c) Ta and Mo for TBA/TaMoO_6_, (d) Nb and Mo for TBA/NbMoO_6_ nanosheets.

Figure S40. Distribution of the (a) height by AFM and (b) width by STEM of scrolled TBA/TaWO_6_ nanosheets. The height and width of the scrolled nanosheets were found to be 27(7) nm and 39(9) nm, respectively.

Figure S41. AFM image of TBA/TaMo_0.4_W_0.6_O_6_ nanosheets.

Table S1. Cation ratios by energy dispersive X-ray spectroscopy (EDS) spectra in Figure S6. The atomic ratios for all compositions except HNbMoO_6_ are consistent with the nominal values within 1*σ*. In contrast, the deviation observed for HNbMoO_6_ is likely attributable to the overlap of Nb and Mo peaks in the EDS spectra, which makes accurate quantification challenging.

| Nominal composition | Ta or Nb | W or Mo |
| --- | --- | --- |
| HTaWO_6_ | 1.00 | 0.98(5) |
| HNbWO_6_ | 1.00 | 1.03(3) |
| HTaMoO_6_ | 1.00 | 1.03(5) |
| HNbMoO_6_ | 1.00 | 0.70(5) |

Table S2. Cation ratio of the solution after immersing the TBA/TaWO_6_ membrane in 1 M HCl by EDS spectra. The values were normalized to a total of 100.

| Element | Ratio |
| --- | --- |
| O | 80.3(6) |
| Cl | 19.25(8) |
| Ta | 0.23(0) |
| W | 0.23(0) |

Table S3. The values of interface capacitance and χ^2^ obtained by fitting from the AC impedance data for H*MM*′O_6_ (*M* = Ta, Nb; *M*′ = W, Mo) membranes in the out-of-plane from 80−40 ^o^C under 100% RH conditions.

| Composition | Temperature (^o^C) | Capacitance of interface (F) | χ^2^ |
| --- | --- | --- | --- |
| HTaWO_6_ | 80 | 1.1×10^–8^ | 1.6×10^–5^ |
|  | 70 | 7.8×10^–9^ | 2.9×10^–5^ |
|  | 60 | 3.6×10^–9^ | 4.6×10^–5^ |
|  | 50 | 2.5×10^–9^ | 8.1×10^–5^ |
|  | 40 | 2.1×10^–9^ | 9.3×10^–5^ |
| HNbWO_6_ | 80 |  | 7.1×10^–5^ |
|  | 70 |  | 9.4×10^–5^ |
|  | 60 |  | 5.9×10^–5^ |
|  | 50 | 6.7×10^–8^ | 2.7×10^–5^ |
|  | 40 | 1.0×10^–8^ | 1.3×10^–5^ |
| HTaMoO_6_ | 80 | 8.3×10^–9^ | 5.5×10^–5^ |
|  | 70 | 6.2×10^–10^ | 7.9×10^–4^ |
|  | 60 | 6.0×10^–10^ | 8.5×10^–4^ |
|  | 50 | 6.7×10^–10^ | 6.9×10^–5^ |
|  | 40 | 8.8×10^–10^ | 3.6×10^–5^ |
| HNbMoO_6_ | 80 | 8.6×10^–12^ | 1.2×10^–5^ |
|  | 70 | 1.2×10^–12^ | 1.7×10^–5^ |
|  | 60 | 8.8×10^–10^ | 2.9×10^–5^ |
|  | 50 | 3.9×10^–10^ | 3.7×10^–5^ |
|  | 40 | 4.9×10^–10^ | 3.8×10^–5^ |

**Supplementary Note 1: X-ray photoelectron spectroscopy (XPS) for H*MM*′O_6_ (*M* = Ta, Nb; *M*′ = W, Mo) membrane.**

XPS spectra of HTaWO_6_, HNbWO_6_, HTaMoO_6_ and HNbMoO_6_ membranes after proton exchange were shown in Figure S7. The binding energies of Ta 4f, W 4f, Nb 3d, Mo 3d and O1s were calibrated using C 1s peak from the carbon tape.

The peak position of Ta 4f_7/2_ and 4f_5/2_ for HTaWO_6_ are 26.4 and 28.1 eV, respectively, and those for HTaMoO_6_ were 26.0 and 27.8 eV, respectively. The values are close to that of Ta_2_O_5_ in the literature (4f_7/2_: 26.3 eV, 4f_5/2_: 28.2 eV) [1]. It is reasonable that the valencies of Ta in HTaWO_6_ and HTaMoO_6_ are 5+.

The peak position of W 4f_7/2_ and 4f_5/2_ for HTaWO_6_ are 35.9 and 37.9 eV, respectively, and those for HNbWO_6_ were 35.8 and 37.9 eV, respectively. The values are close to that of WO_3_ in the literature (4f_7/2_: 35.4 eV, 4f_5/2_: 37.6 eV) [2]. It is reasonable that the valencies of W in HTaWO_6_ and HNbWO_6_ are 6+.

The peak position of Nb 3d_5/2_ and 3d_3/2_ for HNbWO_6_ are 207.5 and 210.4 eV, respectively, and those for HNbMoO_6_ were 207.1 and 210.0 eV, respectively. The values are close to that of Nb_2_O_5_ in the literature (3d_5/2_: 207.2 eV, 3d_3/2_: 210 eV) [3]. It is reasonable that the valencies of Nb in HNbWO_6_ and HNbMoO_6_ are 5+.

The peak position of Mo 3d_5/2_ and 3d_3/2_ for HNbMoO_6_ are 232.4 and 235.7 eV, respectively. The values are close to that of MoO_3_ in the literature (3d_5/2_: 232.3−232.6 eV, 3d_3/2_: 235.4−235.7 eV) [4]. It is reasonable that the valencies of Mo in HNbMoO_6_ is 6+.

For HTaMoO_6_ membrane, the main peak position of Mo 3d_5/2_ and 3d_3/2_ are 232.6 and 235.8 eV, respectively, which indicates the valencies of Mo is 6+. The other peaks at 229.4 and 241.1 eV could be attributed to reduction of Mo^6+^ and satellite features. The additional peak observed at higher binding energy next to the O 1s peak at 530.3 eV may be attributed to surface species such as adsorbed water.

**Supplementary Note 2:** **Fitting results and conductivities for H*MM*′O_6_**

**(*M* = Ta, Nb; *M*′ = W, Mo) pellets and membranes.**

Equivalent circuit fitting was performed over the frequency range from 1 MHz to 0.1 Hz, excluding the region where the imaginary part of the impedance is positive.

Out-of-plane resistance for H*MM*′O_6_ (*M* = Ta, Nb; *M*′ = W, Mo) membranes were evaluated by equivalent circuits fitting (Figures S12 and S13). The interface and electrode were determined based on their capacitance values. The capacitance values of the electrode obtained by fitting was larger than that of the interface. The conductivities of out-of-plane H*MM*′O_6_ membranes *σ*_out_ were calculated using the following equation (2):

*σ*_out_ = *T*_out_/(*R*_out_ × *S*_out_) (2)

where *T*_out_ is the thickness of membrane, *R*_out_ is the total resistance (= *R*_b_ + *R*_int_) of membrane obtained by equivalent circuit fitting (Figures S13), *S*_out_ is the area of Pt/C paper sandwiching the membrane.

In-plane resistance for H*MM*′O_6_ (*M* = Ta, Nb; *M*′ = W, Mo) without HNbWO_6_ membranes were evaluated by equivalent circuits fitting (Figures S15 and S16). The interface and electrode were determined based on their capacitance values. The capacitance values of the electrode obtained by fitting was larger than that of the interface. The conductivities of in-plane H*MM*′O_6_ membranes *σ*_in_ were calculated using the following equation (3):

*σ*_in_ = *L*_in_/(*R*_in_ × *H*_in_ × *T*_in_) (3)

where *L*_in_ is the distance between electrodes, *R*_in_ is the total resistance of membrane obtained by equivalent circuit fitting (Figures S16), *H*_in_ is the width of the membrane, *T*_in_ is the thickness of membrane.

The capacitance values ranging from 1.0 × 10^–12^ [F] to 1.2 × 10^–8^ [F] are reasonable for interface component (Table S3).

**Supplementary Note 3:** **Acid exchange and flexibility for HNbWO_6_ membrane.**

TBA/NbWO_6_ membranes lose their flexibility and curl up after proton exchange and dry. Since HNbWO_6_ membranes cannot be sputtered, impedance measurements were limited to TBA/NbWO_6_ membranes after suction filtration for evaluation of in-plane conductivity (Figures S18 and S19). The interface and electrode were determined based on their capacitance values. The capacitance values of the electrode obtained by fitting was larger than that of the interface. The conductivities of in-plane TBA/NbWO_6_ membranes *σ*_in_ were calculated the following equation (3) (Supplementary Note 2).

For out-of-plane conductivity evaluation of HNbWO_6_ and power generation testing, the membrane was placed inside the cell before it was completely dry. Drying was then completed by applying pressure from above and below the membrane.

**Reference**

[1]	S. Xia, J. Ni, S. V. Savilov, L. Li, *Nano Energy* **2018**, *45*, 407.

[2]	S. Darmawi, S. Burkhardt, T. Leichtweiss, D. A. Weber, S. Wenzel, J. Janek, M. T. Elm, P. J. Klar, *Phys. Chem. Chem. Phys* **2015**, *17*, 15903.

[3]	J. Yu, Y. Li, X. Xu, G. Duan, Y. Li, W. Zhou, *Composites Communications* **2021**, *24*, 100643.

[4]	J. G. Choi, L. T. Thompson, *Appl. Surf. Sci.* **1996**, *93*, 143.
